# Lifecycle Completion and Reproductive Improvement of *Chrysoperla carnea* (Stephens) (Neuroptera: Chrysopidae), Following a Prey Shift Routine During Larval Development

**DOI:** 10.3390/biology14010010

**Published:** 2024-12-26

**Authors:** Muhammad Waleed Shakoor, Jawwad Hassan Mirza, Muhammad Kamran, Fahad Jaber Alatawi

**Affiliations:** Department of Plant Protection, College of Food and Agriculture Sciences, King Saud University, Riyadh 11451, Saudi Arabia; waleedm406@yahoo.com (M.W.S.); jmirza@ksu.edu.sa (J.H.M.); kamran1513@gmail.com (M.K.)

**Keywords:** pupal development, larval development, biocontrol, pest management, natural prey, *Ephestia cautella*

## Abstract

The present study aimed to assess the lifecycle completion and reproductive improvement of green lacewing, *Chrysoperla carnea* (Stephens), larval instars in a prey shift routine while alternatively feeding on motiles of two-spotted spider mite, *Tetranychus urticae* Koch (natural prey), and the eggs of almond moth, *Ephestia cautella* (Walker) (factitious prey). The results showed that *C*. *carnea* larvae successfully completed their lifecycle and life span in the treatments where either the 2nd (T2) or 3rd (T3) instar larvae were fed with the eggs of *E*. *cautella* while other instars were provided with motiles of *T*. *urticae*, i.e., 1st–3rd and 1st–2nd, respectively. The predator larvae failed to transform from pupae to adults when only the 1st instar larvae were fed on the *E*. *cautella* eggs (T1). Similar results were found when all predator instars were reared on the motiles of *T*. *urticae* (T4). The shortest mean total larval duration and preoviposition period, the highest fecundity and fertility, and balanced sex ratio were recorded when 3rd instar larvae were fed only on *E*. *cautella* eggs (T3). The longest adult longevity and oviposition period were recorded when only the 2nd instar larvae were fed on *E*. *cautella* eggs (T2). The findings of the present study would be helpful in developing a self-sustained mite biological control strategy using *C*. *carnea* and provide a roadmap for future studies to elucidate the larval release efficiency of *C*. *carnea* with the supplementary diet for the management of greenhouse mite pests.

## Simple Summary

The insect generalist predator, *Chrysoperla carnea*, is an important biological control agent for different soft-bodied insects and mite pests. It has been reported on different occasions that *C*. *carnea* larvae could not complete development when only feeding on mite motiles. The present study aimed to follow a prey shift routine while alternating the provision of almond moth eggs and motiles of two-spotted spider mites. These were provided to different larval instars of the predator in different treatments. The results showed that providing the eggs of the almond moth to only the 2nd or 3rd instar larvae of the predator provided enough nutrients for the predator’s developmental completion and reproductive success. These findings are useful for developing a successful biological control program against mites with the substitutive use of a supplementary diet.

## 1. Introduction

The green lacewing, *Chrysoperla carnea* (Stephens) (Insecta: Neuroptera: Chrysopidae), is a voracious predator of a wide range of soft-bodied insects and mite pests of different agricultural crops and orchards [1,2,3,4,5,6,7].

The biology of *C. carnea* has been studied, feeding on a variety of insect pests [5,6,7,8,9,10]. It has been elucidated that the prey on which *C. carnea* larvae feed could affect the predator’s life cycle [11]. As a generalist predator, *C. carnea* develops and reproduces successfully, feeding on various insect species with high nutritional content as food sources [5,7,9,12,13].

Among the mite pests, the two-spotted spider mite (TSSM), *Tetranychus urticae* (Koch), (Acari: Prostigmata: Tetranychidae) is one of the major cosmopolitan mite pests, causing economic damage to a wide range of host plants in greenhouses and fields [14,15,16,17]. Studies of *C*. *carnea* feeding on mite pests are scarce. Mostly, the functional responses and feeding efficiency of this predator have been reported only against *T*. *urticae* [6,7,18,19]. However, it has been reported that *C. carnea* larvae could not complete their life cycle and transform into adults when reared on motiles of *T*. *urticae* [7,12,20]. Contrastingly, only Saleh et al. [21] reported that *C*. *carnea* could complete its lifecycle when reared on the females of *T*. *urticae*. In recent experiments conducted in our laboratory, we determined that all three larval instars of *C*. *carnea* consumed a high number of motile stages of two mite species: “date palm mite”, *Oligonychus afrasiaticus* (McGregor), and “citrus brown mite”, *Eutetranychus orientalis* Klein but could not transform from pupae to adults [Shakoor et al. unpublished].

The *C*. *carnea* could be a suitable biocontrol agent for mite pests, but failure to complete its life cycle masked its potential use in mite biological control programs. It was suggested that the life history parameters of *C*. *carnea* could be improved by alternating diet of the predator larval instars [2]. The potential of *C. carnea* for mite management in fields and greenhouses could be enhanced by providing alternate food sources like eggs of almond moth, *Ephestia cautella* (Walker), during some of its larval life spans. The eggs of different lepidopteran pests have been used for the mass rearing of *C*. *carnea* [22,23,24], and the biological parameters of the predator were significantly improved when provided with the eggs of *E*. *cautella* [Shakoor et al. unpublished]. The present research, for the first time, aimed to study the lifecycle completion and reproductive success of the indigenous predator, *C*. *carnea*, by rearing the larvae in a prey shift routine, i.e., alternatively feeding on motiles of *T. urticae* (natural prey) along with supplementary food source: the eggs of *E. cautella*. It is expected that this study could be helpful in the development of successful biological control strategies for spider mite pests using *C*. *carnea* both in open fields and greenhouses.

## 2. Material and Methods

### 2.1. Source of Predator

The adults of the indigenous predator, *Chrysoperla carnea*, were collected from cultivated vegetation and were mass-reared in the laboratory, feeding its larvae on different aphid species and eggs of the almond moth, *Ephestia cautella*. The adults were fed a liquid supplementary diet following UlHaq et al. [25].

### 2.2. Rearing of Prey

The population of prey, two-spotted spider mite (TSSM), *Tetranychus urticae*, was initially acquired from the infested eggplants. The species was identified by the experts at the Acarology Research Laboratory, College of Food and Agriculture King Saud University, Kingdom of Saudi Arabia. The mite’s colony was maintained on small-sized eggplants grown in the greenhouse and used in the experimentation, and the *E. cautella* eggs were obtained from the Biological Control Research and Development Unit (BCRD), College of Food and Agriculture King Saud University, where it has been mass-reared on a mixed poultry and chicken diet.

### 2.3. Experimental Procedure

#### 2.3.1. Prey Host-Shift

The experiment was conducted in a climate-controlled chamber (Memmert^®^, Schwabach, Germany) at 25 ± 2 °C and 65 ± 5% RH, 16:8 L:D photoperiod. Five treatments were performed as explained in Table 1: T1 = 1st instar provided with eggs of *E. cautella*, and 2nd and 3rd instars provided with TSSM motiles, T2 = 2nd instars fed on eggs of *E. cautella*, while TSSM motiles provided to 1st and 3rd instars; T3 = 1st and 2nd instars of the predator fed on TSSM motiles and 3rd instar fed on eggs of *E. cautella*; T4 = Control, all three instars (1st, 2nd and 3rd) were fed on TSSM motiles; T5 = Control, all three instars (1st, 2nd, and 3rd) were fed on *E. cautella* eggs. Each treatment was replicated 20 times.

#### 2.3.2. Prey Provision to Predator Larvae

The small plastic cups (7.5 cm diameter, depth 3 cm) were used as experimental units. Two eggs of *C. carnea* were kept in each cup. After the emergence, only one larva was kept inside each experimental cup. The motiles of *T. urticae* were provided to the predator larvae on a small leaf disc of the eggplant. The *E. cautella* eggs were simply placed in the cup without any substrate. The leaf disc was replaced with a fresh one, and the experimental units were cleaned daily. The larvae completed development and pupated inside the cup. The pupae were transferred singly to plastic jars for the emergence of adults. After emergence, adults were kept singly for two to four days to let them develop physically and to differentiate between the male and female adults before being mated. For the oviposition, females were provided with a black sheet in the jar. The eggs were removed using forceps and transferred to plastic cups to determine their fertility and incubation period.

The developmental duration of each instar (1st, 2nd, and 3rd), total larval duration, pupal incubation period, sex ratio, adult longevity, including pre-oviposition, oviposition, and oviposition period of female, fecundity, fertility, and egg incubation period were recorded at12-h interval during the whole experimental period. In the event that adults did not emerge from the pupa, the respective pupae were dissected to confirm the death of the instar.

### 2.4. Statistical Analysis

The data was analyzed using One-Way ANOVA to test the effect of different prey shift routines on the developmental period of different immature stages, pupal incubation, sex ratio, male and female (pre-oviposition, oviposition, and post-oviposition) adult longevity, fecundity, fertility, and egg incubation of *C. carnea*. Means were separated using Duncan’s Multiple Range Test (DMRT) at *p* < 0.05. Sex ratios were calculated using a Chi-square test (i.e., number of females/number of females + males). All analyses were performed using the SAS computer program version 9.2 (SAS Institute^®^, Cary, NC, USA, 2008) [26].

## 3. Results

The larvae of *Chrysoperla carnea* completed their lifecycle successfully when either 2nd (T2) or 3rd (T3) instar larvae were fed on *E*. *cautella* eggs (Table 2). When only the 1st instar larvae (T1) were fed with eggs of *E*. *cautella*, the pupae failed to emerge as adults (T1). Similar results were found when all instars (T4) were provided with motiles of *T*. *urticae* (Table 2).

There were significant differences found among the treatments for the developmental duration of each larval instar (Table 2). The developmental duration of the 1st, 2nd, and 3rd instar larvae was shorter when fed only with the eggs of *E*. *cautella* in treatments T1–3, respectively. The shortest mean total larval developmental period was recorded in the treatment (T3; 13.19 ± 0.52 days), where only 3rd instar fed on *E*. *cautella* eggs (Table 2) as compared to the control (T4; 19.4 ± 1.56 days). While pupal duration was significantly shorter in the treatment (T2; 8.87 ± 0.41 days) where only the 2nd instar was provided with eggs of *E*. *cautella* (Table 2), in the treatments T1 and T4, all the pupae failed to emerge as adults. Hence, their total developmental period only represents the days the pupae were alive.

There was no significant difference in the longevity of males and females between T2 and T3 compared to control (T5) (Table 3). The sex-ratio was strictly male-biased in treatment T2 and female-biased in T3 and T5 (Table 3).

A longer pre-oviposition period was observed in the adult females when only the 2nd instar larvae were fed on the *E. cautella* eggs (T2, Table 3), while there was no significant difference in the preoviposition period of females when only the 3rd instar larvae were fed on the *E. cautella* eggs as compared to the control (T5, Table 3).

The oviposition period is significantly reduced in treatments T2 and T3 compared to the control (T5) (Table 3). A longer post-oviposition period was recorded when the 2nd and 3rd larval instars were fed on *E*. *cautella* eggs in T2 and T3, respectively, compared to the control treatments (T5). The incubation period of the F1 eggs in treatments T2 and T3 was significantly longer compared to control T5 (Table 3).

## 4. Discussion

This is the first study to evaluate the effect of prey shift on the reproductive improvement and lifecycle completion of *C. carnea*, feeding on motiles of the natural prey, two-spotted spider mite (TSSM), *Tetranychus urticae*, and alternatively feeding on the factitious host, *Ephestia cautella* eggs, under the laboratory conditions.

The successful completion of the life cycle and biology of generalist predators rely on the nutrition they get from the prey during its larval duration [27]. The last two instars of *C*. *carnea* have been reported as crucial stages for predation efficiency and life cycle completion [3,4,5,6,7,28]. The *C*. *carnea* larvae completed the developmental and reproductive period either when the 2nd or 3rd instar larvae were fed on *E*. *cautella* eggs, while the adults failed to emerge from pupae when only the 1st instar larvae were provided with *E*. *cautella* eggs (Table 2). It demonstrates the importance of the nutritional composition of *E*. *cautella* eggs provided to the 2nd and 3rd instars of *C*. *carnea* for the development and life cycle completion. It has been reported that the egg production process (vitellogenesis) could be initiated in the late instars prior to adulthood and is correlated with the nutritional uptake during larval development [27]. Hence, the 2nd and 3rd instar larvae, when fed on the eggs of *E*. *cautella*, resulted in lifecycle completion, unlike when the eggs were provided to only the 1st instar larvae (T1), which was not helpful in providing nutritional requirements to *C*. *carnea* development. Saleh et al. [21], in contrast, reported that the *C*. *carnea* larvae successfully transformed from pupae to adults when fed with females of *T*. *urticae*. It can be argued here that the provision of only immature or all motiles of *T*. *urticae* could have resulted in differential results. The nutritional superiority of females over other motile stages has not been explored yet.

The nutritional suitability of the prey plays a key role in the successful development and reproduction of the predator. The prey/host provided during larval duration affects the reproductive success of the predator *C. carnea* [29,30]. In the present findings, the prey supplied during different larval stages affected the emergence of adults. In the treatment (T1), as well as control (T4), the pupae failed to transform into adults. Kasap et al. [12] reported that while feeding on the motiles of *T. urticae*, the predator was not able to reach adulthood. It could be deduced that *T. urticae* was not nutritionally sufficient to complete the predator life cycle. Studies on *C. carnea* and another chrysopid predator against *T. urticae* have reported mites as low-nutritional prey compared to insect prey hosts [20,31]. This situation does not happen only with natural prey but could also occur when the predator is reared on alternate diets. It has been reported that only 25% of *C. carnea* larvae completed their lifecycle when fed only maize pollen. However, 87% of larvae completed their lifecycle when one instar larvae was fed with pollen while the other two instars were provided with *E*. *kuehniella* eggs [32].

Although the adults, resulting from each successful treatment, were provided with a uniform liquid diet, the differences in the fecundity and eggs’ hatchability in those treatments further prove the effect of food provided during the larval developmental duration on the fecundity and F1 egg’s hatchability. The fecundity and fertility of adults whose 3rd instar larvae fed on *E*. *cautella* eggs (T3) was higher compared to when only the 2nd instar (T2) larvae were fed on the eggs (Table 3). In contrast, the females had shorter oviposition periods in T3 compared to T2. It proves that the developmental and reproductive success of the *C*. *carnea* depends upon the dietary nutrition provided to the 3rd instar. As mentioned earlier, it could be due to the initiation of egg production during this instar [27]. Interestingly, the developmental period of the 3rd instar while feeding on *E*. *cautella* eggs did not differ from when its prior two instars developed feeding on mites (Table 2). It reflects that the 3rd instar took longer to replenish developmental energy, which the previous instar was unable to get [27]. It definitely resulted in reduced total larval duration in T3 and higher female reproductive parameters than in T1 and T2 (Table 3).

The prey’s nutritional value considerably affects the development of the predator [33]. As found in the present study, the motiles of *T. urticae*, which may be nutrient deficient, did not meet the nutritional requirement at the 2nd and 3rd instar, resulting in failed pupation. However, it has been reported that the number of mite individuals consumed was exponentially high, favoring the nutritional inferiority of mites for the *C. carnea* development [20,21,34,35] (Shakoor et al. unpublished). Feeding on prey with high nutrition value results in a shorter generation time and higher reproductive potential of the predator. The nutritional superiority of eggs over the later instars of the same species has been reported [36] (Shakoor et al. unpublished data). *Chrysoperla carnea* larvae fed more of the eggs of *Corcyra cephalonica* as compared to its larvae, and its larval development period was shorter while feeding on eggs as compared to larvae of *C. cephalonica* [36]. Similarly, the recent laboratory experiment depicted that *C*. *carnea* larvae successfully developed into adults while feeding on eggs of citrus brown mite, *Eutetranychus orientalis*, while the larvae failed to pupate when fed on motiles of the same species [Shakoor et al. unpublished data].

The field application of *C*. *carnea* for mite management has been rarely studied [18,37]. The predator was reported unsuccessful in providing management of *T*. *urticae* infesting papaya in the field [19]. Together with the findings of the present study, it is indistinct that *C*. *carnea*, as a generalist, would tend to search for more nutritional food options in the field. The results of the present study provide an obsolete solution where the predator should be provided with eggs of *E*. *cautella* on the crop where the mite infestation occurs. In this way, mite management would be achieved because the voraciously feeds on mites, and its nutritional deficiency to complete the life cycle would be achieved by feeding on eggs of *E*. *cautella*.

In the current findings, the extended larval feeding during the 2nd and 3rd instars, as compared to the 1st instar of the predator, plays a key role in the completion of the life cycle of the predator *C. carnea*. A substantial impact of host shift was evident, leading to increased fecundity and hatchability when the 2nd and 3rd instars were provided with *E. cautella* eggs, showing a correlation with the control where the highest fecundity and hatchability rates are recorded. It was observed that as the predator larvae pass through the later instars, they undergo significant physiological development, enabling them to consume larger quantities of prey. This extended feeding duration allows for greater accumulation of energy reserves and nutrients essential for the subsequent stages of development, including pupation and metamorphosis into adulthood [15]. Additionally, prolonged feeding during the later instars enabled optimal growth and development of adults capable of effective reproduction [38]. The findings signify the effect of prey host shift on the biology of *C. carnea* during the larval instar period. It emphasizes that the nutritional suitability of the prey host should be considered for the successful implications of the generalist predator *C. carnea*. These findings may contribute to a better understanding of the relationship between prey nutrition and predator biology, which is inevitable for the effective implementation of biocontrol pest management practices. The recommendations could be devised for the efficient field release of *C*. *carnea* larvae supplemented with the eggs of factitious hosts for the sustained management of mite pests. Future studies may expand the scope of greenhouse and field trials to ensure the success of such release strategies.

## 5. Conclusions

This study provides the first comprehensive assessment of how prey shifts impact the reproductive improvement and life cycle completion of *C*. *carnea* when its larval instars were alternatively fed on *T*. *urticae* motiles and *E*. *cautella* eggs. The results highlight that feeding during the 2nd and 3rd instar larvae on *E*. *cautella* eggs is critical for the successful development and reproduction of *C*. *carnea*. The nutritional quality of the prey played a key role in ensuring the completion of the predator’s life cycle. In addition to that, *T. urticae* motiles prove insufficient nutrition for pupation and adult emergence of the *C*. *carnea*. However, the improved fecundity and hatchability observed in treatments where later instars were fed with *E*. *cautella* eggs suggested that a nutrient-rich diet during key developmental stages significantly increases reproductive performance. These findings emphasized the importance of considering prey nutritional suitability for using generalist predators like *C*. *carnea* in biological control strategies. Future research should focus on evaluating the field applications of this approach to enhance pest management outcomes.

## Figures and Tables

**Table 1:** Prey shift routine for the provision of eggs of almond moth, *Ephestia cautella* (Walker), and motiles of two-spotted spider mite, *Tetranychus urticae* Koch, alternatively to larval instars of the indigenous predator, *Chrysoperla carnea* (Stephens), in each treatment.

Treatments	Prey Provided to Each Larval Instar of the Predator		
	1st Instar	2nd Instar	3rd Instar
**T1**	*E. cautella* eggs	*T. urticae* motiles	*T. urticae* motiles
**T2**	*T. urticae* motiles	*E. cautella* eggs	*T. urticae* motiles
**T3**	*T. urticae* motiles	*T. urticae* motiles	*E. cautella* eggs
**T4** (Control)	*T. urticae* motiles	*T. urticae* motiles	*T. urticae* motiles
**T5** (Control)	*E. cautella* eggs	*E. cautella* eggs	*E. cautella* eggs

**Table 2:** Mean ± SE larval and pupal duration of the indigenous predator, *Chrysoperla carnea*, under the effect of prey shift against two-spotted spider mites, *Tetranychus urticae* Koch and almond moth, *Ephestia cautella* (Walker).

Treatment	Larval Instars Duration			Mean Total Larval Duration	Pupae
	1st Instars	2nd Instars	3rd Instars		
**T1** **(E-M-M)** *	3.12 ± 0.13 c	3.72 ± 0.16 c	11.63 ± 1.36 a	18.5 ± 1.65	8 ± 0.00 b (Failed to emerge)
**T2 (M-E-M)**	4.17 ± 0.14 a	2.71 ± 0.12 d	12.13 ± 1.40 a	19.01 ± 1.66	8.87 ± 0.41 b
**T3 (M-M-E)**	4.32 ± 0.11 a	4.16 ± 0.12 b	4.71 ± 0.29 b	13.19 ± 0.52	9.7 ± 0.12 a
**T4** (Control) **(M-M-M)**	3.65 ± 0.14 b	5.05 ± 0.20 a	10.70 ± 1.22 a	19.4 ± 1.56	10 ± 0.00 a (Failed to emerge)
**T5** (Control) **(E-E-E)**	2.85 ± 0.08 c	2.47 ± 0.09 d	3.10 ± 0.35 b	8.42 ± 0.52	9.97 ± 0.15 a
	DF 4,94; F 25.94; *p* < 0.001	DF 4,87; F 51.32; *p* < 0.001	DF 4,75; F 17.46; *p* < 0.001	-	DF 4,38; F 5.67; *p* = 0.0011

The treatment means followed by the same letter in a column are not significantly different. * In the treatment column, “**E**” stands for *E. cautella* eggs, and “**M**” stands for the motiles of *T. urticae* provided to the instars, and their arrangement shows the specific instar to which they were provided.

**Table 3:** Mean ± SE adult longevity, reproductive period, fecundity, hatchability, and egg incubation duration of indigenous predator, *Chrysoperla carnea* (Stephens), under the effect of prey shift against two-spotted spider mite, *Tetranychus urticae* Koch, and almond moth, *Ephestia cautella* (Walker).

Treatment	Adults’ Longevity (Days)		Sex-Ratio (M: F%)	Pre-Oviposition (Days)	Oviposition (Days)	Post-Oviposition (Days)	Fecundity (Number)	Hatchability (Number)	Incubation Duration
	Male	Female							
**T1** (**E-M-M**) *	-	-	-	-	-	-	-	-	-
**T2** **(M-E-M)**	30.40 ± 2.08 a	35.00 ± 0.00 a	0.14	4.00 ± 0.00 a	18.00 ± 0.00 b	10.00 ± 0.00 ab	140.00 ± 0.00 b	108 ± 0.00 b	4.28 ± 0.10 a
**T3** **(M-M-E)**	27.28 ± 2.61 a	32.60 ± 3.91 a	0.58	2.20 ± 0.20 b	16.20 ± 1.11 b	13.50 ± 0.86 a	181.00 ± 1.60 b	149 ± 1.52 b	4.45 ± 0.02 a
**T4** (Control) **(M-M-M)**	-	-	-	-	-	-	-	-	
**T5** (Control) **(E-E-E)**	29.00 ± 1.00 a	37.22 ± 0.54 a	0.60	1.88 ± 0.20 b	22.22 ± 0.43 a	8.44 ± 0.74 b	293.22 ± 1.54 a	240.77 ± 1.53 a	3.80 ± 0.06 b
	DF 2,16; F 0.56; *p* = 0.58	DF 2,12; F 1.26; *p* = 0.32	-	DF 2,12; F 6.58; *p* = 0.01	DF 2,12; F 18.86; *p* = 0.0002	DF 2,11; F 7.91; *p* = 0.007	DF 2,12; F 37.94; *p* < 0.001	DF 2,12; F 32.10; *p* < 0.001	DF 2,12; F 26.08; *p* < 0.001

The treatment means followed by the same letter in columns are not statistically different by Duncan’s multiple range test (*p* < 0.05). * In the treatment column, “**E**” stands for *E. cautella* eggs, and “**M**” stands for the motiles of *T. urticae* provided to the instars, and their arrangement shows the specific instar to which they were provided. Sex ratio = number of females/number of females plus males.

## Data Availability

All necessary data are provided in this paper.
